# Supplementary material for: Mandarin fish (Sinipercidae) genomes provide insights into innate predatory feeding
Source: Commun Biol. 2020 Jul 9;3:361. doi: 10.1038/s42003-020-1094-y (PMC7347838; doi:10.1038/s42003-020-1094-y)
Supplement: Supplementary file 1 — Supplementary Information [file 42003_2020_1094_MOESM1_ESM.pdf]

Supplementary Methods

Processing of sequencing data

Nextera mate pair Illumina reads were screened for Nextera and Illumina adaptor sequences and trimmed accordingly by NextClip v1.3.2[1]. The paired-end Illumina data had a tight insert size distribution around 300 bp and could be used without prior processing due to the high kmer assembly and correction steps during IDBA-UD assembly[2]. Rare adaptor sequences left in the data did not hinder the assembly. They were too short to result in valid read overlaps with large kmer sizes. Pacific biosciences SMRT raw reads were corrected and trimmed by the CANU v1.0 assembler[3].

Cost efficient reference assisted *de novo* assembly of *S. kneri, S. scherzeri* and *C. whiteheadi* based on short read sequencing

Our short reads genome assemblies relied on only two short read sequencing libraries, which can be obtained as highly complex libraries (low numbers of PCR duplicates) in most labs without expert knowledge on long distance library construction:
A) 2*150 bp paired end library (300bp insertsize); seq. cov. about 20×
B) 2*150 bp “gel-free” Nextera mate pair library (wide distribution of insertsizes, Peak ~3Kbp); seq. cov. about 20×
Short read data for *S. kneri*, *S. scherzeri* and *C. whiteheadi* was *de novo* assembled in a hybrid approach involving the kmer based *de Bruijn* graph assembler IDBA-UD and the overlap-layout-consensus (OLC) based NEWBLER v3.0 (Roche) assembler. We used a tweaked version of IDBA-UD allowing for kmers up to 252 nt and readlength of up to 300bp. We first assembled the Illumina paired end datasets (2*150bp, ~20×) using the following parameters:
--mink 54; --maxk 252; --seed_kmer 60; --similar 0.97; --no_coverage.
The resulting contigs were duplicated to produce a 2× coverage kmer backbone for the assembly of the mate pair data, which was then assembled in a second iteration of idba_ud:
-l “2xassembled_contigs.fa” --mink 206; --maxk 252; --step 20; --no_coverage; --similar 0.98; --seed_kmer 60
The resulting contigs and locally assembled contigs (local-contigs) of the different kmer iterations were splitted at unknown “n” bases and, if exceeding the NEWBLER V3.0 readlength limit, splitted into fragments of 29.000 bp with 4.000bp overlap. These splitted contigs were treated as “pseudo longreads” during the NEWBLER assembly.
To overcome the disadvantages of the broad distribution of insert sizes in Nextera gelfree mate pair library preparations, the mate pair data was size selected “*in-silico*”. This means the reads were mapped (BWA MEM) against the sinChu7 reference genome and written to fastq files of distinct size classes. Mate pairs that could not be mapped consistently were written into a file with unknown size distribution. The mate pair data (reverse complemented orientation = FR is of importance for efficient scaffolding in this regard) was added to the NEWBLER assembler to produce a scaffolded OLC assembly.
To minimize misassemblies, scaffolds were checked for synteny by aligning them against the sinChu7 reference assembly and scaffolds were splitted at detected putative inter-chromosomal fusions. Finally, we ordered the scaffolds by alignment to the sinChu7 reference genome and applying RAGOUT, performed gap closure using Platanus gap_close of all data[4] and joined neighbouring contigs, if overlaps were detectable. The derived assemblies were called sinKne6, sinSch6 and corWhi6.

Repeat annotation

We used RepeatModeler v1.0.8 for *de novo* analysis of repeat sequences in the genome assembly sinChu7, sinKne6, sinSch6 and corWhi6. The resulting repeat sequence libraries were applied with RepeatMasker v4.0.6 to annotate/mask repetitive sequences in sinChu7, sinKne6 sinSch6 and corWhi6[5,6].

Gene model prediction in *S. chuatsi* by protein homology and evidence from RNAseq

We performed a homology based coding sequence (cds) prediction on the sinChu7 genome assembly first. We downloaded 1.765.230 Proteins assigned to Teleostei from the NCBI RefSeq database (date of download 03.05.2017). The proteins were aligned to the genome assembly by SPALN v2.06f to result in cds models for sinChu7 (parameters: -M4 -t 12 -O0 -Q7 -LS -pq)[7,8]. As SPALN sometimes outputs cds models that harbor early stop codons, we re-calculated all ORFs using the TRANSDECODER (https://transdecoder.github.io/). The corrected genomic cds coordinates were converted to gtf format and combined into non-redundant cds models using TACO[9]. If a locus had several alternative models, the model with the longest cds sequence was chosen as representative gene model.

Subsequently, we used HISAT2[10] to align RNAseq data from brain, muscle, liver and gut samples of *S. chuatsi* against the genome assembly. We supported HISAT2 mapping by adding a database of potential splice sites from the homology based cds prediction. STRINGTIE v1.2.3[11] was used to assemble the mapped RNAseq reads into transcript models. RNAseq transcript models and homology based CDS models were combined by TACO and TRANSDECODER was used to assign genomic cds- and UTR-exon coordinates to the resulting transcript models.

Annotation of gene function

For functional annotation we aligned protein sequences of the sinChu7 gene models against 4 fish species protein datasets that are relatively well annotated (RefSeq annotation: *Lates calcarifer*; ENSEMBL annotation: *Oreochromis niloticus*, *Gasterosteus aculeatus* and *Danio rerio*). We extracted the protein sequences of up to 100 best scoring matches per sinChu7 protein (some gene families have many similar scoring matches, it is hard to assign orthology), created multiple sequence alignments by MAFFT[12] and calculated a phylogenetic tree for each sinChu7 protein (FASTTREE2[13]). Gene descriptions and symbols were assigned from the protein match that had the smallest branch length distance to the sinChu7 protein (in most cases the gene description was assigned from the RefSeq annotation of the closely related *L. calcarifer*, while gene symbols were taken from the Ensembl annotations as they were not available for most *L. calcarifer* RefSeq proteins).

Transfer of gene annotation from sinChu7 to sinKne6, sinSch6 and corWhi6

SPALN v2.06f[7,8] was applied for spliced alignment of sinChu7 annotated mRNAs and corresponding proteins to the genome assemblies sinKne6, sinSch6 and corWhi6, respectively. The resulting mRNA and cds models were combined using TACO[9]. After calling the ORFs by TRANSDECODER (https://transdecoder.github.io/), the highest scoring transcript model per gene (score ~ cds length) was chosen as the reference gene model.

We assigned gene description and symbols from sinChu7 functional annotation and estimated orthology by micro synteny.

Whole genome alignment

Whole genome alignments were performed by LAST aligner and lastsplit[14]. MAF output files were converted to psl format using the maf-convert script. Blocks of shared collinearity between a pair of genomes were filtered by custom scripts (chaining local alignments, if they had consistent distances and orientation in both genomes in two iterations; iteration 1 maximum distance difference of blocks in both genomes 2,500 bp; iteration 2 removing blocks with length lower than 12,000 bp after iteration 1 and re-calculating blocks with maximum distance difference of 100,000 bp) using sinChu7 as the reference coordinates. If blocks of collinearity showed rearrangements, we calculated link coordinates (Fig. 1). The block and link coordinates were visualized by CIRCOS[15].

Supplementary Figures

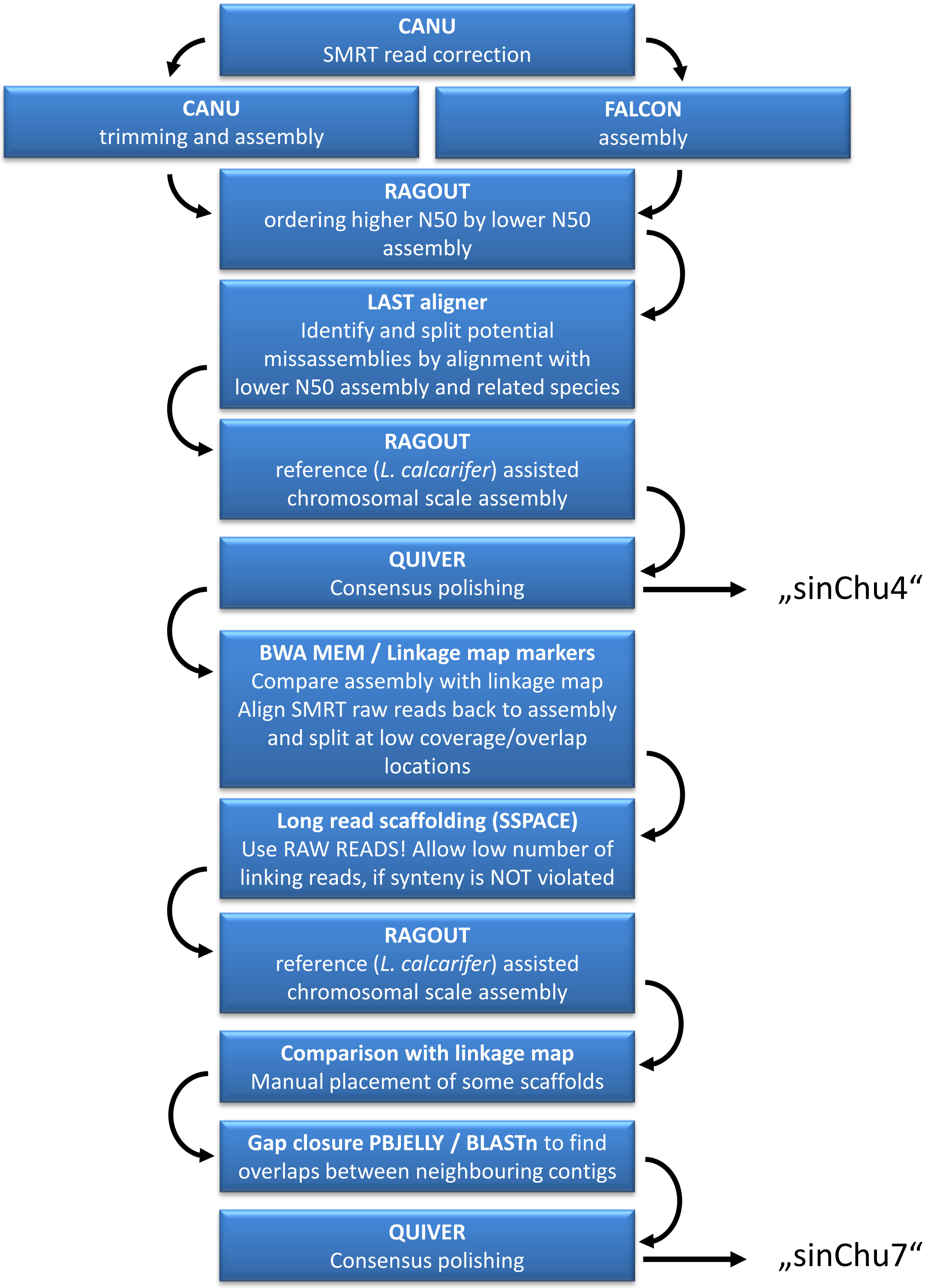

Supplementary Fig. 1 Overview of the bioinformatic pipeline for assembling the *S. chuatsi* genome. sinChu: *Siniperca chuatsi.*

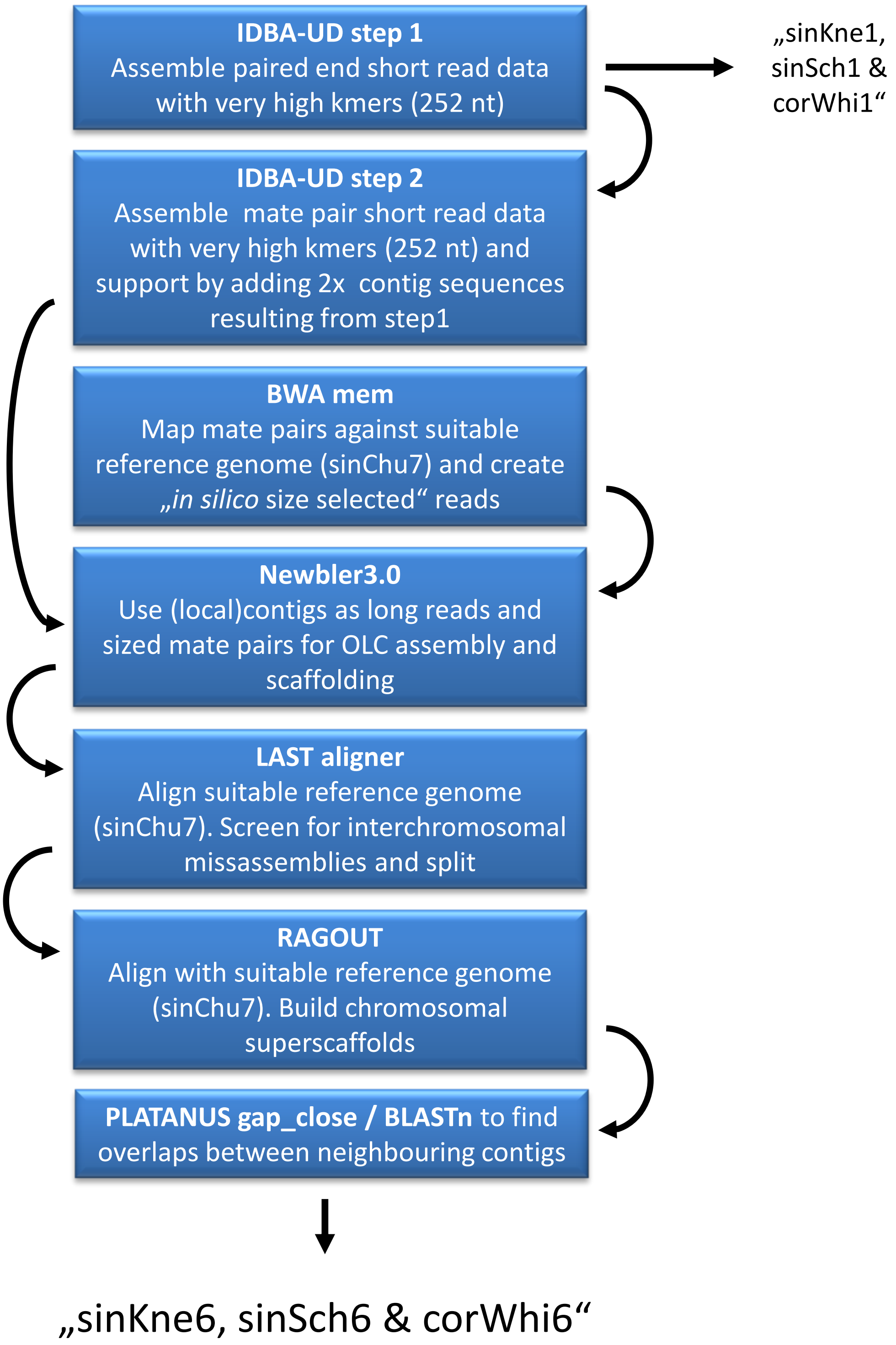

Supplementary Fig. 2 Overview of the pipeline for assembling *S. kneri, S. scherzeri and C. whiteheadi* genomes. sinKne: *Siniperca kneri*, sinSch: *Siniperca scherzeri*, corWhi: *Coreoperca whiteheadi*.

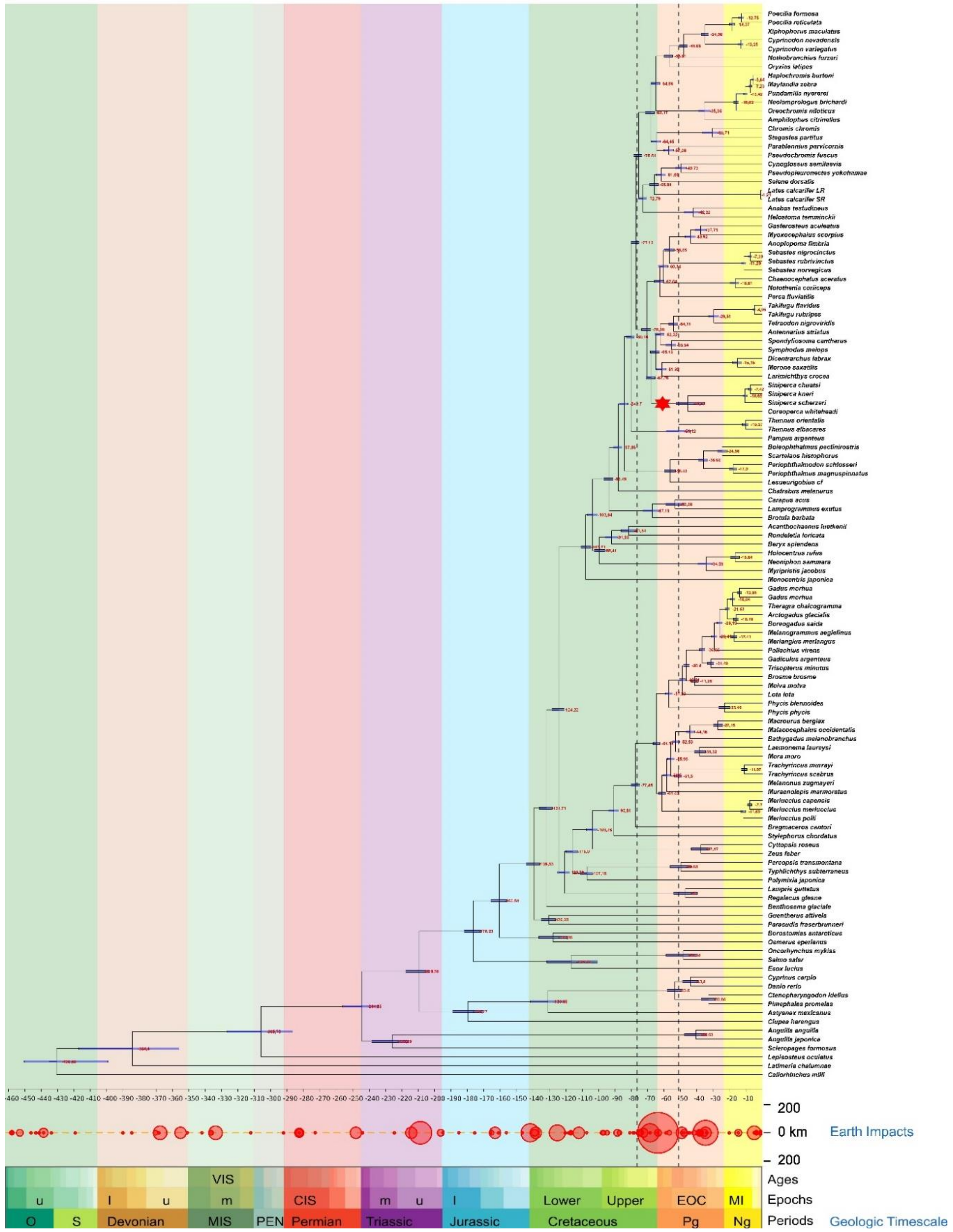

Supplementary Fig. 3 Time-calibrated phylogenomic tree calculated from noncoding portions of whole-genome alignments. The SH-aLRT support was 100 for all branches except one, which had a value of 98. Divergence times (red or x-axis) were estimated by MCMCTree[16] (clock=2 model) using a few calibration timepoints from www.timetree.org. The mandarin fish (Sinipercidae) clade is indicated with "✸".

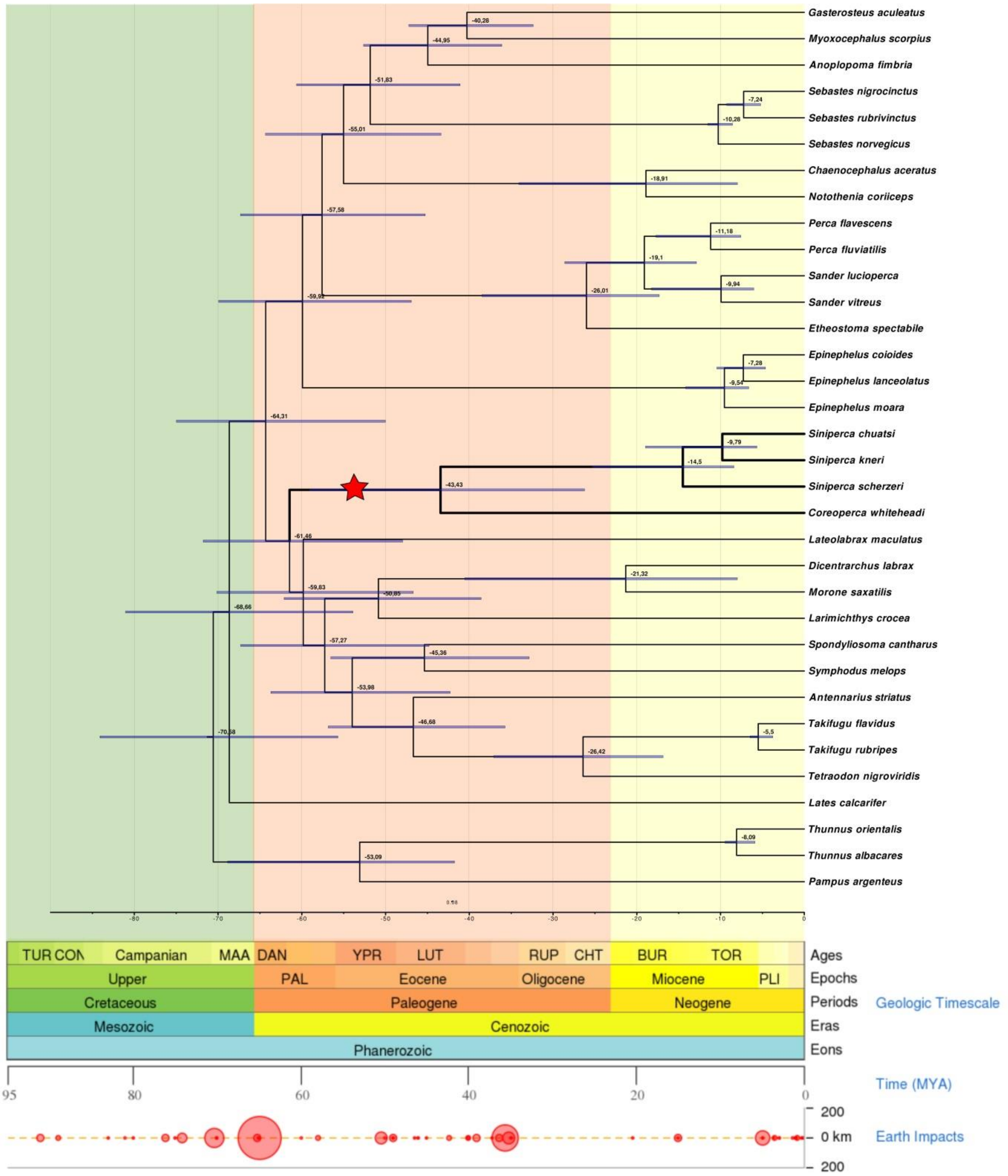

Supplementary Fig. 4 Time-calibrated phylogenomic tree calculated from noncoding portions of whole-genome alignments. The SH-aLRT support was 100 for all branches. Divergence times (red or x-axis) were estimated by MCMCTree[16] (clock=2 model) using a few calibration timepoints from www.timetree.org. The mandarin fish (Sinipercidae) clade is indicated with "★".

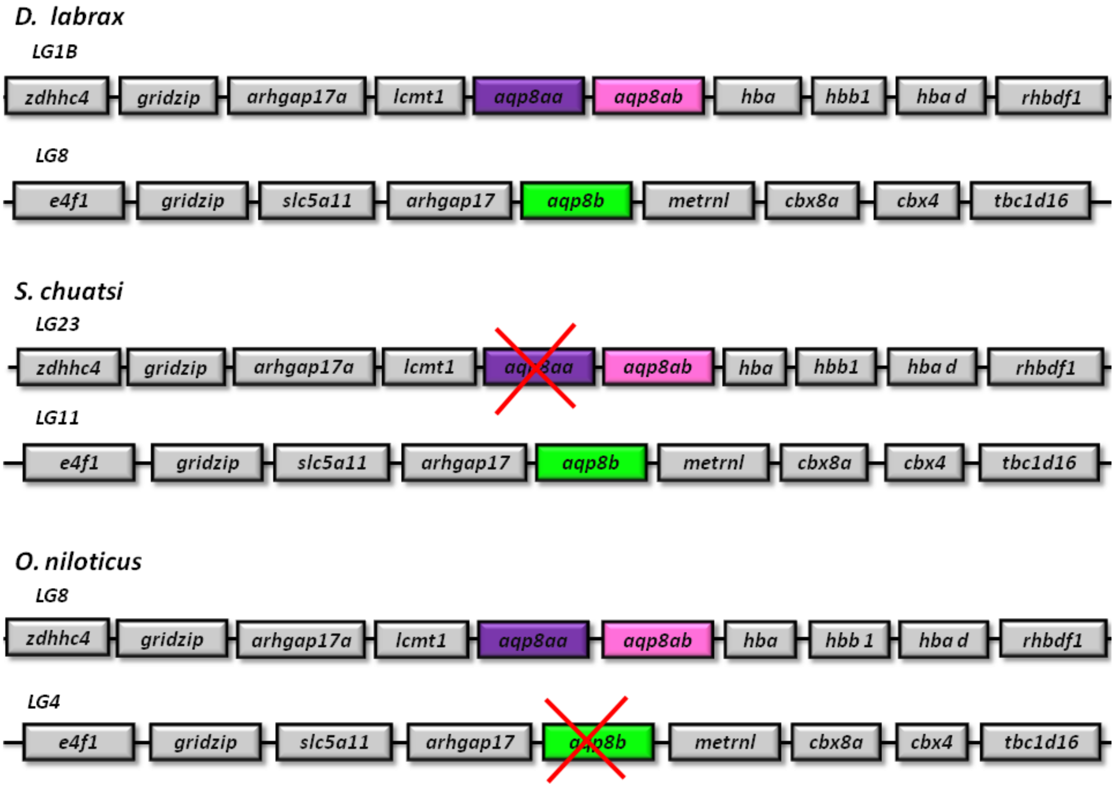

Supplementary Fig. 5 Synteny analysis of *aqp8* genes in *D. labrax*, *S. chuatsi* and *O. niloticus*. Synteny analysis was performed by searching flanking gene(s) of *aqp8s* using ensemble genome browser (http://www.ensembl.org/index.html), UCSC Genome Bioinformatics (http://genome.ucsc.edu/index.html) and Map Viewer (http://www.ncbi.nlm.nih.gov/mapview/).

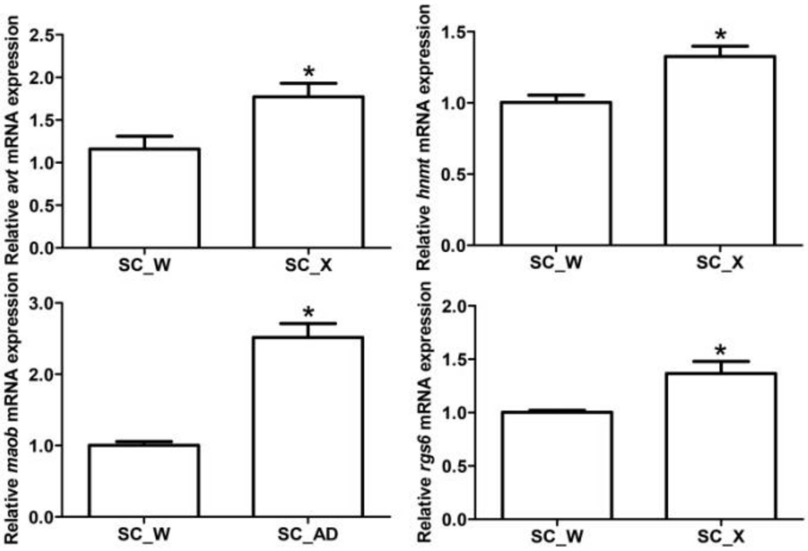

Supplementary Fig. 6 Validation of differentially expressed genes in nonfeeders (SC_W) and feeders (SC_X or SC_AD) with RT-QPCR. Brain tissues were used for analysis[17]. The amplification efficiencies and sequence information of primers were listed in Supplementary Table 14. Gene expression levels were quantified relative to the expression of *rpl13a* using the optimized comparative Ct ($2^{-\Delta\Delta Ct}$) value method[18]. Data were presented as means ± SEM (n = 6). *indicated significant difference ($P < 0.05$).

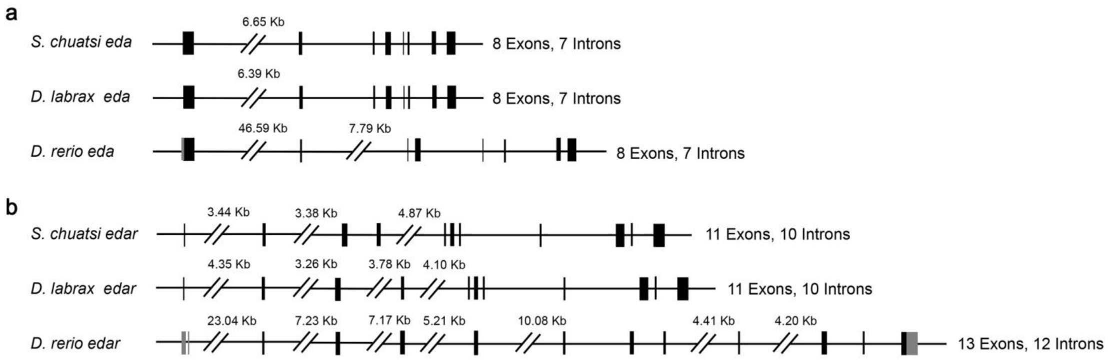

Supplementary Fig. 7 Genomic structures of *eda* and *edar* in *S. chuatsi*, *D. labrax* and *D. rerio*. The black and gray boxes indicate exons. The gray boxes represent 5'-UTR and 3'-UTR respectively.

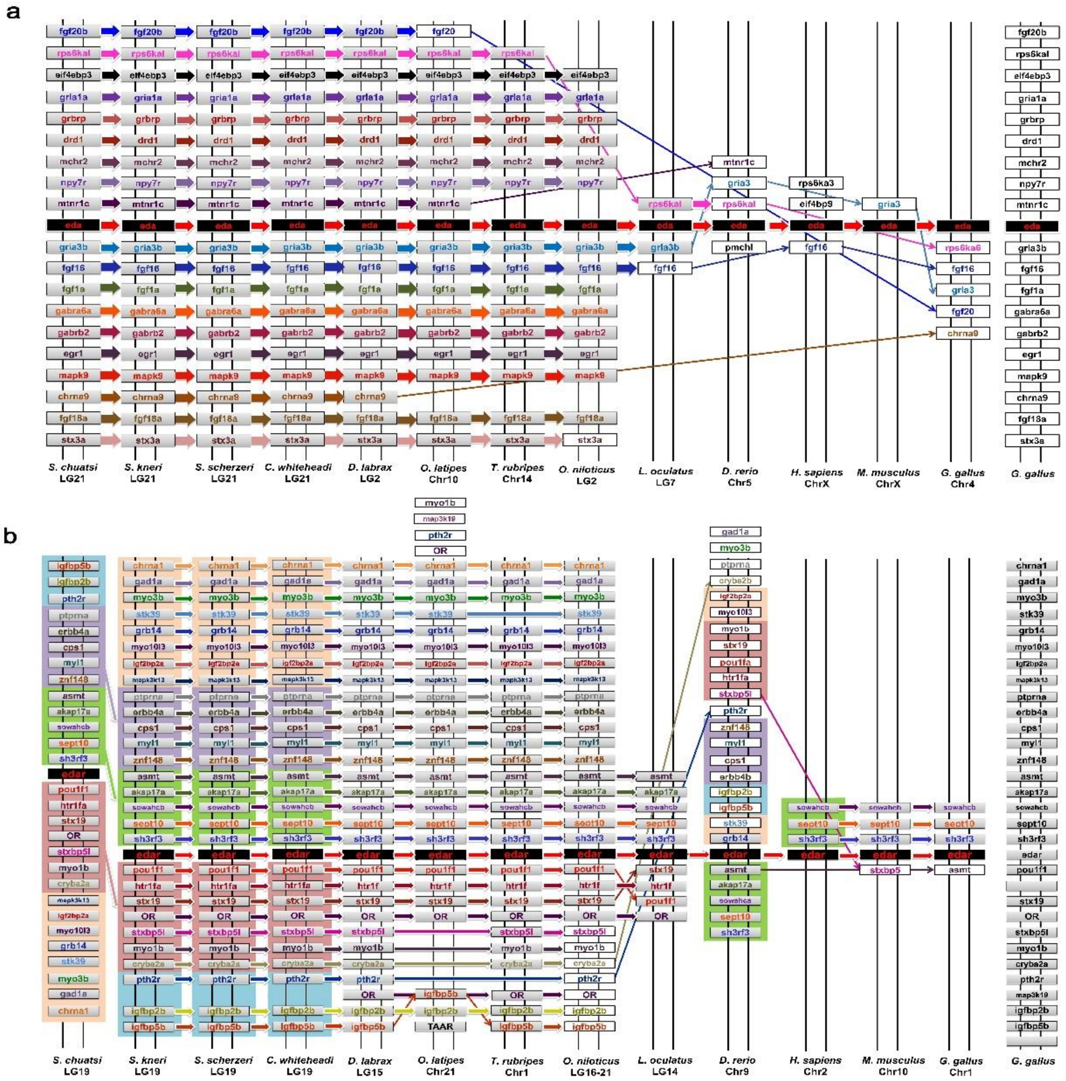

Supplementary Fig. 8 Synteny analysis of *eda* (a) and *edar* (b). *Eda* and *edar* genes show conserved synteny across vertebrates.

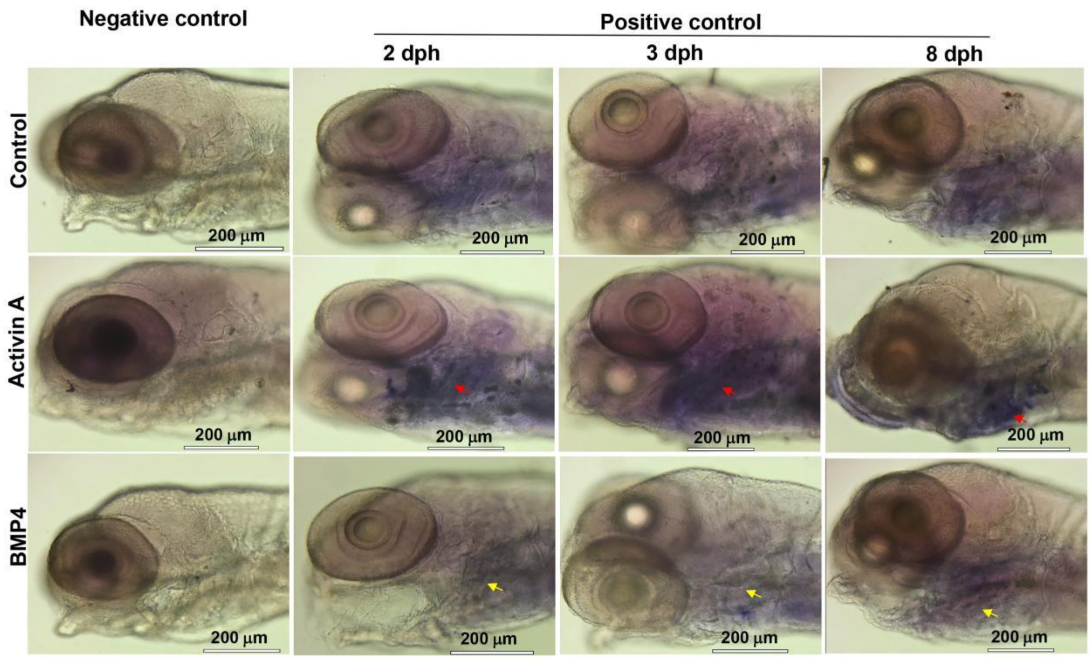

Supplementary Fig. 9 Zebrafish *edar* expression assessed by whole mount *in situ* hybridization. Higher expression of *edar* (red arrowhead) were observed in the gill of fish treated with 1 ng/ml Activin A (*edar* activator), and lower expression (yellow arrowhead) were observed in fish treated with 50 ng/ml BMP4 (*edar* inhibitor).

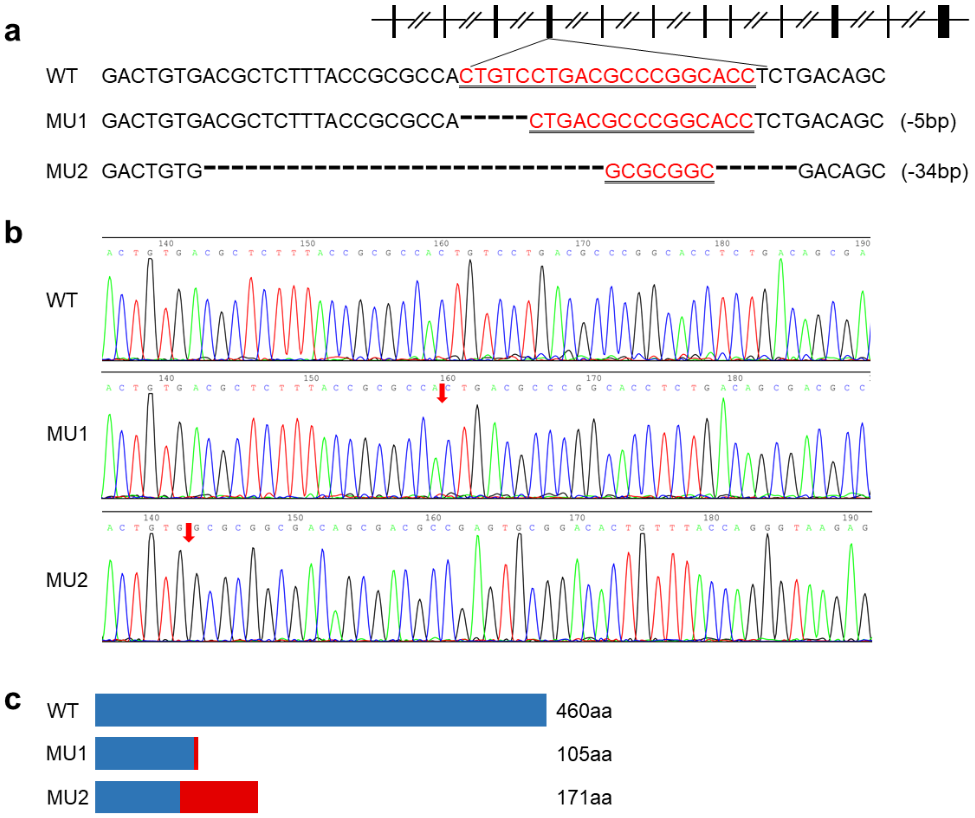

Supplementary Fig. 10 Generation of *edar* knockout zebrafish. a The target site was underlined in red font. WT: wild type; MU1 and MU2: two mutants with 5bp and 34bp deletion, respectively. **b** Sequencing maps of WT, MU1 and MU2. The deletions in MU1 and MU2 were indicated by red arrows. **c.** Predicted amino acids of EDAR, blue rectangles were identical to WT EDAR, red rectangle indicated miscoding amino acids.

Supplementary Tables

Supplementary Table 1. Chromosomal/superscaffold only assembly statistics.

Assembly	sinChu7	sinKne6	sinSch6	corWhi6
**Quality**	"nearly finished"	HQ draft	HQ draft	HQ draft
**DATA and approx. seq. coverage**	SMRT ~50X	Illumina PE/MP ~30X	Illumina PE/MP ~30X	Illumina PE/MP ~40X
**superscf. Count**	24	34	28	34
**superscf. length [bp]**	730,055,992	714,256,286	722,311,289	691,332,895
**Gap length [bp]**	1,076,089	19,263,755	22,157,228	18,224,152
**N50 superscf. length [bp]**	30,577,383	29,890,789	30,166,107	28,748,119
**N50 superscf. count**	11	11	11	11
**average superscf. length [bp]**	30,419,000	21,007,538	25,796,832	20,333,320
**largest superscf. [bp]**	38,234,813	37,751,638	38,358,931	36,907,144
**placed contig count**	328	19,070	19,531	19,143
**placed contig length [bp]**	728,979,903	694,992,531	700,154,061	673,108,743
**N50 placed contig length [bp]**	12,304,016	78,643	85,698	78,820
**N50 placed contig count**	20	2,623	2,385	2,366
**average placed contig length [bp]**	2,222,500	36,444	35,848	35,162
**largest placed contig [bp]**	30,084,615	553,821	739,181	671,346
**assembled nucleotides in chr./superscf.**	96.68%	98.76%	98.18%	97.07%

sinChu: *Siniperca chuatsi*, sinKne: *Siniperca kneri*, sinSch: *Siniperca scherzeri*, corWhi: *Coreoperca whiteheadi*.

Supplementary Table 2. Chromosomal/superscaffold + all unplaced scaffolds statistics

Assembly	sinChu7	sinKne6	sinSch6	corWhi6
**Quality**	"nearly finished"	HQ draft	HQ draft	HQ draft
**DATA and approx. seq. coverage**	SMRT ~50X	Illumina PE/MP ~30X	Illumina PE/MP ~30X	Illumina PE/MP ~35X
**scf. count**	1,156	1,854	2,826	2,615
**scf. length [bp]**	755,061,740	723,605,295	736,220,003	712,478,531
**Gap length [bp]**	1,078,130	19,895,218	23,119,548	
**N50 scf. length [bp] *incl. Map/Synteny***	30,508,166	29,777,865	30,166,107	28,603,870
**N50 scf. Count *incl. Map/Synteny***	12	12	11	12
**N50 scf. length [bp] *no Map/Synteny***	23,370,180	1,198,238	1,381,907	1,020,541
**average scf. length [bp]**	653,168	390,294	260,517	272,458
**largest scf. [bp]**	38,234,813	37,751,638	38,358,931	36,907,144
**contig count**	1,464	21,467	23,070	22,717
**contig length [bp]**	753,983,610	703,710,077	713,100,455	693,395,931
**N50 contig length [bp]**	12,191,788	77,505	83,589	76,435
**N50 contig count**	21	2,679	2,461	2,495
**average contig length [bp]**	515,016	32,781	30,910	30,523
**largest contig [bp]**	30,084,615	553,821	739,181	671,346

sinChu: *Siniperca chuatsi*, sinKne: *Siniperca kneri*, sinSch: *Siniperca scherzeri*, corWhi: *Coreoperca whiteheadi*.

Supplementary Table 3. Positively selected genes triggering enrichment of GO/MP terms related to observed biological traits

Traits	Branches involved in analysis	Total number of PSGs with assigned gene symbol	Identified GO/MP related to traits	Number of PSGs	p-value for enrichment	Genes triggering GO/MP term enrichment
**Feeding habit**	Branch 3 and *S. scherzeri*	544	learning or memory	20	7.30E-03	Abca7, B4galt2, Cacna1e, Cic, Dnah11, Ehmt2, Hif1a, Itga3, Itga8, Itpr3, Lamb1, Map1a, Ntan1, Ntrk1, Pak6, Plcb1, Psen2, Ptn, Ptprz1, Sorcs3
			locomotion	102	2.00E-07	Abi3, Abl1, Ace, Adgrb1, Adgrl3, Aire, Amotl2, Ano6, Anxa1, Ash1l, Bves, Cacna1e, Ccr8, Cd99, Cd99l2, Cdc42bpb, Cdh1, Cdh13, Cep131, Chrm4, Cmklr1, Crk, Csf1r, Dcc, Depdc1b, Dlc1, Dlg5, Dnah11, Dock2, Ednra, F2rl1, Fbln1, Fer, Fgfbp1, Flrt2, Flt1, Gli3, Hdac5, Hif1a, Hoxa7, Inpp5b, Ist1, Itga2, Itga2b, Itga3, Itga4, Itga6, Itgb8, Lama5, Lamb1, Lemd3, Lmo4, Lrp6, Magi2, Mia3, Myo10, Myo9b, Mypn, Nfasc, Nipbl, Nisch, Notch1, Ntrk1, Pard3, Pcsk5, Phldb2, Pik3c2b, Pik3cd, Pik3cg, Pkn1, Plcb1, Pld1, Plxnb1, Ppp2r3a, Prok2, Psen2, Ptgdr2, Ptk2b, Ptk7, Ptn, Ptpn22, Ptprc, Ptprj, Ptpro, Ptprz1, Rreb1, Rsph9, Ryk, Scg2, Sema4a, Sema4d, Sema7a, Sep, 2Sod2, St14, Stk10, Sun2, Tie1, Trim25, Trim35, Ttn, Vstm2l
	S. scherzeri	334	response to food	3	3.30E-02	Bcl10, Slc25a25, Prkcg
	Branch 3 (PSG p<0.005)	86	eating behaviour	2	3.10E-02	Lepr, Uchl3
**Growth**	*S. chuatsi* and *S. kneri*	411	growth	42	2.30E-02	Agrn, Apba2, Brd4, C3, Cdkn1b, Cgref1, Chd7, Cobl, Ctc1, Egfr, Ep300, Fn1, Gli3, Golga4, Hoxa11, Ifrd1, Kmt2c, Kmt2d, Lepr, Matn2, Mbd5, Med12, Nek1, Pou1f1, Ppm1f, Ppt1, Ptprj, Rims1, Rmi1, Rps6kb1, Sema4b, Shank3, Sirt1, Socs7, Spr, Supv3l1, Tlr2, Tmem108, Tns2, Trpc5, Wasf1, Wnt3
			decreased circulating insulin-like growth factor I level	8	1.10E-03	Arid1b, Itprid2, Mbd5, Sirt1, Slc38a3, Spr, Suco, Tut4
			decreased growth hormone level	5	9.90E-03	Lepr, Mbd5, Pou1f1, Sirt1, Zfhx3
	S. kneri	206	proportional dwarf	3	7.80E-03	Nek1, Pou1f1, Wdr62
**Pyloric caeca**	Branch 5 and *C. whiteheadi*	1687	alimentary system mucosa	123	7.00E-07	Abca1, Abcf1, Acaa2, Acsl5, Adamts9, Aebp1, Ahnak, Ano8, Ano9, Apod, Aqp3, Arhgap29, Atp1b1, Axin2, Bmp7, Boc, Cast, Ccdc80, Cdcp1, Cdx4, Cep83, Chga, Col18a1, Col1a1, Col1a2, Col2a1, Col4a1, Col4a2, Col4a4, Col4a5, Col4a6, Crabp1, Creb3l3, Cxcr4, Cyld, Cyp26a1, Dll4, Dnah12, Dsc2, Dsg2, Dsp, Dst, Dtna, Ecm1, Eln, Epas1, Epcam, Esrp2, Esyt3, Ets1, Exo1, F11r, Fbln1, Fgf10, Fgf4, Fgfr1, Fn1, Foxe1, Foxn1, Gfpt1, Gsn, Has2, Hip1r, Hnf4a, Hnf4g, Hs6st3, Hspa8, Hspg2, Igf2r, Igsf9, Irx1, Irx3, Irx5, Isl1, Itga2, Itga3, Itga4, Itga6, Itm2a, Jup, Krt15, Krt5, Lama3, Lama5, Lamb1, Lamb2, Lamb3, Mapkbp1, Matn4, Mmrn2, Mpzl2, Ncoa6, Nedd8, Nid1, Notch2, Oma1, Otx2, Pik3ip1, Pls1, Plxnb1, Plxnb2, Ppargc1a, Prom2, Qsox1, Rab20, Rara, Rarb, Rif1, Robo2, Shroom2, Smad3, Smad4, Smad6, Snai1, Sod3, Sox6, Spint2, Spo11, St14, Stard10, Syt1, Tacc1, Tbx22, Tfec, Tgfbr2, Thbd, Tle3, Tmprss15, Tox3, Trim24, Usp22, Vav3, Wnt4
			digestive/alimentary phenotype	164	0.198	Abca1, Acan, Ace2, Actn4, Acvrl1, Adam10, Adgrg7, Aebp1, Ahcyl1, Aicda, Angpt2, Aqp3, Aqp4, Arhgap29, Atf4, Bcam, Bmp7, Bnc2, Capn6, Capn8, Casp3, Cbx2, Ccdc39, Ccdc80, Ccnd1, Cd36, Cd63, Cgn, Chst11, Chuk, Cldn18, Cntn1, Col11a1, Col19a1, Col1a1, Col2a1, Col4a2, Col4a4, Col7a1, Crb3, Ctnnd1, Cxcr4, Cyld, Cyp26a1, Dcc, Dhcr7, Dsp, Dst, Dyx1c1, Elf3, Enpp7, Epcam, Eya1, F2r, Fadd, Fads2, Fam20c, Fen1, Fga, Fgf10, Fgfr1, Fgfr3, Foxc1, Foxe1, Foxf2, Galnt3, Gcgr, Gcnt4, Gfra2, Gli2, Golgb1, Gon4l, Gpc3, Hsd17b4, Hspg2, Ift88, Il2rg, Il6st, Isl1, Kif1bp, Krt15, Krt5, Lama3, Lama5, Lamb3, Ldb3, Lig4, Lrp2, Ltbp4, Lzts1, Map3k14, Midn, Mmp9, Mnt, Ncoa6, Nod2, Nr2f2, Nr4a2, Nrtn, Nsun2, Otx2, P2ry4, Pcnt, Pcsk5, Pcsk6, Pdgfra, Pex1, Pglyrp2, Pkd1, Plau, Pls1, Porcn, Ppargc1a, Prdm14, Ptgs1, Pth1r, Qsox1, Rab34, Rb1cc1, Rbpj, Relb, Ripk3, Rrm2b, Runx2, Sc5d, Sdccag8, Serpinf1, Shroom3, Slc10a2, Slc15a1, Slc18a2, Slc27a4, Slc2a12, Slc32a1, Slc5a2, Slc5a7, Slc6a19, Slc8a1, Smad3, Smad4, Socs1, Sptlc2, Sstr2, St14, Stard10, Supv3l1, Sytl2, Tbc1d32, Tbx22, Tctn2, Tdp2, Tent5c, Tgfbr2, Thbd, Tln1, Tlr2, Tm6sf2, Tnfrsf11a, Tnfrsf1a, Tnk1, Ttc7, Ulk4, Usp22, Wdr19, Wfs1, Xpnpep1
**Salinity adaptation**	Branch 7	918	ion transport	127	6.80E-05	Abca4, Abcb11, Ada, Ahnak, Ank3, Ano5, Ano6, Ano7, Apoa1, Apoa4, Apoc2, Apoe, Aqp8, Arg1, Asic1, Atp13a3, Atp1a2, Atp4b, Atp6v0a2, Atp6v1e1, Atp8b5, Bin1, Cacna1a, Cacna1c, Cacna1d, Cacna1f, Cacna1g, Cacng6, Cacng7, Ccs, Cd36, Chchd10, Chrna2, Chrna7, Chrna9, Chrnd, Clcc1, Cln3, Cnga1, Cpt1b, Cracr2b, Cyba, Cybb, Enpp1, Fgf12, Gabra6, Gabrg3, Gpr39, Hcn2, Hcn3, Homer2, Htr2a, Ip6k2, Kcna1, Kcna2, Kcna7, Kcnab3, Kcnh7, Kcnip2, Kcnj15, Kcnk18, Mif, Nfatc1, Nnt, Nsf, Opa1, Oprd1, Oxsr1, P2ry2, Pcp4, Pkd1, Plin2, Pllp, Prkca, Prkg2, Psap, Psen1, Ptk2b, Rbp4, Rhag, Saraf, Scn1b, Scn4a, Scn8a, Sestd1, Shank3, Slc10a1, Slc12a6, Slc13a2, Slc15a1, Slc16a1, Slc20a1, Slc23a2, Slc24a2, Slc25a4, Slc27a2, Slc30a7, Slc37a2, Slc39a14, Slc39a3, Slc39a7, Slc39a8, Slc44a1, Slc4a1, Slc4a11, Slc5a2, Slc6a1, Slc6a2, Slc6a5, Slc6a6, Slc9a1, Slc9a3, Stac2, Steap4, Stxbp1, Tgfb1, Tgfb2, Tmc2, Tmem163, Tmem30b, Tmem63a, Tnfrsf11a, Tnfsf11, Trpm2, Trpv4, Vdr, Wfs1
			cellular hypotonic response	4	4.41E-04	Oxsr1, Prkg2, Slc12a6, Trpv4
**Aggression**	*S. chuatsi*	212	abnormal aggression-related behavior	5	2.20E-02	Hydin, Lgals3, Ppt1, Ptchd1, Shank3
	Siniperca clade	1528	abnormal emotion/affect behavior	87	0.07	Adcyap1r1, Adgrl3, Adora2a, Akap11, Alk, Als2, Apba2, Arhgap32, Arhgap44, Arid1b, Arsg, Atg7, Atp1a3, C3, Cacna1b, Cacna1e, Cers6, Cntnap2, Crhbp, Csf1r, Cyfip1, Dact2, Dlgap2, Drd1, Dst, Ehmt1, Ehmt2, Eno2, Erc2, Fmr1, Frmd4b, Gcnt4, Gdpd5, Grm1, Grm8, Hdc, Hgsnat, Hnf4g, Htr1b, Hydin, Idua, Itpr3, Jup, Kcnb2, Kif13a, Lgals3, Lzts1, Magi2, Map6, Mbd5, Mov10, Mpp5, Mto1, Nav2, Ncor1, Notch3, Npy2r, Nt5e, Ntan1, Ntrk1, Opa1, P2rx5, Per3, Ppp1r2, Ppp1r26, Ppt1, Prkcg, Ptchd1, Ptgfrn, Ryr3, Shank3, Slc17a5, Slc17a6, Slc18a2, Slc5a7, Socs7, Stx1a, Tlr3, Trpc2, Trpc5, Trpm2, Uba6, Ube3a, Ubr1, Ulk2, Wasf1, Wfs1

Supplementary Table 4. Positively selected and differentially expressed genes between nonfeeders and feeders in Node 3

SC7 gene_ID	Gene symbol	Description	BMP4-initiated signaling molecules binding sites
**SC7-LG05_06038**	*nfatc3a*	nuclear factor of activated T-cells, cytoplasmic 3-like	
**SC7-LG07_10081**	*fam160b2*	protein FAM160B2-like isoform X3	
**SC7-LG08_10806**	*akap9*	A-kinase anchor protein 9	
**SC7-LG09_12591**	*hif1al*	hypoxia-inducible factor 1-alpha-like	
**SC7-LG16_20555**	*ly75*	lymphocyte antigen 75-like	
**SC7-LG17_21189**	*agmo*	alkylglycerol monooxygenase	Xvent-1
**SC7-LG20_25777**	*si:ch1073-396h14*	disintegrin and metalloproteinase domain-containing protein 10-like isoform X1	
**SC7-LG20_25889**	*vtg1*	vitellogenin-like isoform X3	
**SC7-LG22_28752**	-	uncharacterized protein LOC108891332 isoform X2	
**SC7-LG23_29008**	*abcc3*	canalicular multispecific organic anion transporter 2 isoform X1	
**SC7-LG23_29346**	*prodh2*	probable proline dehydrogenase 2	
**SC7-LG24_30448**	*slc22a13*	solute carrier family 22 member 13 isoform X3	

Supplementary Table 5. Positively selected and differentially expressed genes between nonfeeders and feeders in *S. scherzeri*

SC7 gene_ID	Gene symbol	Description	BMP4-initiated signaling molecules binding sites
**SC7-LG01_01389**	*si:ch73-233k15*	uncharacterized protein LOC108888582	
**SC7-LG02_01949**	-	uncharacterized protein LOC108885782 isoform X5	
**SC7-LG02_02163**	*cnnm1*	metal transporter CNNM1	
**SC7-LG02_02680**	*ncoa4*	nuclear receptor coactivator 4 isoform X1	
**SC7-LG03_04139**	*trim25*	E3 ubiquitin/ISG15 ligase TRIM25-like isoform X5	
**SC7-LG03_04242**	*mtmr7b*	myotubularin related protein 7b	
**SC7-LG05_06904**	*ighmbp2*	DNA-binding protein SMUBP-2	Smad3
**SC7-LG06_08127**	*dvl2*	segment polarity protein dishevelled homolog DVL-2	
**SC7-LG08_11320**	*asns*	asparagine synthetase	Xvent-1
**SC7-LG09_11590**	*ulk2*	serine/threonine-protein kinase ULK2	
**SC7-LG09_11707**	*rbp2a*	retinol-binding protein 2	
**SC7-LG10_12878**	*tfe3a*	transcription factor E3-like	
**SC7-LG13_16974**	*slc25a25b*	calcium-binding mitochondrial carrier protein SCaMC-2-like isoform X1	
**SC7-LG13_17803**	*zgc:171965*	proteinase-activated receptor 1-like	
**SC7-LG15_19104**	*bx548028*	-	
**SC7-LG18_23562**	*pard3*	partitioning defective 3 homolog isoform X10	
**SC7-LG22_28439**	*rnf14*	E3 ubiquitin-protein ligase RNF14 isoform X1	
**SC7-LG23_29911**	-	kinesin-like protein KIF20A isoform X3	Xvent-1
**SC7-LG24_30940**	-	zinc finger protein 708-like	
**SC7-LG02_02157**	-	uncharacterized protein C10orf12-like isoform X1	

Supplementary Table 6. Genes species-specific to *S. chuatsi* and differentially expressed between big-size and small-size group

SC7 gene ID	Gene symbol	Description	BMP4-initiated signaling molecules binding sites
**SC7-LG01_00687**	*ighv1-2*	immunoglobulin heavy variable 1-2	
**SC7-LG01_00731**	*ighv5-3*	immunoglobulin heavy variable 5-3	Xvent-1
**SC7-LG08_10356**	*clec4e*	c-type lectin domain family 4 member e	Xvent-1
**SC7-LG09_12253**	*casr*	extracellular calcium-sensing receptor	
**SC7-LG11_15157**	*igic1s1*	immunoglobulin light iota constant 1	
**SC7-LG12_16403**	*ccl4l*	c-c motif chemokine 4 like	
**SC7-LG16_19863**	*muc5ac*	mucin 5 subtype ac	
**SC7-LG16_20812**	*b3galt2*	beta-1,3-galactosyltransferase 2	
**SC7-LG18_23132**	*muc2*	mucin 2e	
**SC7-LG23_29695**	*cd79b*	□□cell antigen receptor complex-associated protein beta chain	

Supplementary Table 7. Positively selected and differentially expressed genes between big-size and small-size group in *S. chuatsi*

SC7 gene ID	Gene symbol	Description	BMP4-initiated signaling molecules binding sites
**SC7-LG06_07835**	*col4a6*	collagen-6 (IV) chain	
**SC7-LG08_11253**	*paqr7a*	membrane progestin receptor-b	
**SC7-LG06_08332**	*dnah2*	dynein heavy chain 2, axonemal	
**SC7-LG12_16478**	*lgals3b*	galectin 3	Xvent-1
**SC7-LG20_25596**	*hmha1b*	histocompatibility (minor) ha-1b	
**SC7-LG21_27349**	*myot*	myopalladin	Xvent-1
**SC7-LG22_28067**	*ubash3ba*	ubiquitin-associated and sh3 domain-containing protein b	

Supplementary Table 8. Positively selected in *S. kneri* and differentially expressed genes between big-size and small-size group in *S. chuatsi*

SC7 gene ID	Gene symbol	Description	BMP4-initiated signaling molecules binding sites
**SC7-LG01_00815**	*zp3*	zona pellucida sperm-binding protein 3	Xvent-1
**SC7-LG02_01964**	*ggps1*	geranylgeranyl pyrophosphate synthase	
**SC7-LG02_02003**	*capn8*	calpain-2 catalytic subunit	
**SC7-LG04_05182**	*ccnd3*	g1/s specific cyclin d3	
**SC7-LG04_05407**	*kif21b*	kinesin like protein kif21b	
**SC7-LG05_06336**	*mical2b*	molecule interacting with CasL 2	Xvent-1
**SC7-LG07_08943**	*aacs*	acetoacetyl-CoA synthetase	
**SC7-LG10_13957**	*krt5*	keratin 5	
**SC7-LG12_16138**	*efhc1*	ef-hand domain containing protein 1	
**SC7-LG13_17161**	*sh3bp2*	sh3 domain-binding protein 2	
**SC7-LG13_17294**	*slc46a2*	thymic stromal cotransporter homolog	
**SC7-LG13_17920**	*myo18b*	unconventional myosin-XVIIIb	
**SC7-LG14_18276**	*iqcb1*	iq calmodulin binding motif containing protein 1	
**SC7-LG15_18918**	*plekha5*	pleckstrin homology domain containing family a member 5	
**SC7-LG17_21022**	*muc1*	mucin 1	
**SC7-LG18_22838**	*magel2*	mage like protein 2	Xvent-1
**SC7-UN_11_31167**	*hla-dpa1*	hla class II histocompatibility antigen, dp alpha 1 chain	
**SC7-UN_329_32169**	*muc2*	mucin 2	

Supplementary Table 9. Positively selected genes in *C. whiteheadi*

SC7 gene ID	Gene symbol	Description	BMP4-initiated signaling molecules binding sites
**SC7-LG01_00371**	*mcm5*	minichromosome maintenance complex component 5	Smad3, Smad4
**SC7-LG01_00372**	*gcat*	2-amino-3-ketobutyrate coenzyme A ligase, mitochondrial	Xvent-1, Smad4
**SC7-LG01_00380**	*mchr1b*	melanin-concentrating hormone receptor 1-like	Xvent-1, Smad4
**SC7-LG01_00382**	*bptf*	nucleosome-remodeling factor subunit BPTF-like	Xvent-2, Smad3, Smad4

Supplementary Table 10. The number of *pepsin A*, *trypsin* genes and pyloric caeca in mandarin fish

Species	Number of intact *pepsin A*	Number of *pepsin A* pseudogenes	Number of pyloric caeca
Siniperca chuatsi	3	0	117~323
Siniperca kneri	3	0	62~100
Siniperca scherzeri	3	0	65~124
Coreoperca whiteheadi	2	1	3

Supplementary Table 11. The number of Na^+/K^+-ATPase α-1 in selected fish species

Species	Salinity	*atp1a1*	*atp1a2*	*atp1a3*	*atp1b1*	*atp1b2*	*atp1b3*	*atp1b4*	total no.
Cyprinodon variegatus	**SW**	1	1	2	2	1	2	1	10
Stegastes partitus	**SW**	2	1	2	2	2	2	1	12
Dicentrarchus labrax	**SW/BW**	2	1	2	2	2	2	1	12
Larimichthys crocea	**SW/BW**	2	1	3	2	2	1	1	12
Cynoglossus semilaevis	**FW/BW/SW**	1	2	2	2	2	2	1	12
Oreochromis niloticus	**FW/BW/SW**	4	1	2	2	2	2	1	14
Lates calcarifer	**FW/BW/SW**	2	1	2	2	2	2	1	12
Takifugu rubripes	**FW/BW/SW**	2	1	2	2	2	2	1	12
Oryzias latipes	**FW/BW**	3	1	2	1	2	2	1	12
Danio rerio	**FW**	6	1	2	2	2	2	1	16
Astyanax mexicanus	**FW**	6	1	2	2	2	2	1	16
Esox lucius	**FW**	6	1	2	2	1	2	1	15
Siniperca chuatsi	**FW**	2	1	2	2	2	2	1	12
Siniperca kneri	**FW**	2	1	2	2	2	2	1	12
Siniperca scherzeri	**FW**	2	1	2	2	2	2	1	12
Coreoperca whiteheadi	**FW**	2	1	2	2	2	2	1	12

FW: freshwater, BW: brackish water, SW: sea water.

Supplementary Table 12. The number of *aqp8* in selected fish species

Species	Salinity	*aqp8aa*	*aqp8ab*	*aqp8b*	total
Larimichthys crocea	**SW/BW**	1	1	1	3
Lates calcarifer	**SW/BW/FW**	1	1	1	3
Dicentrarchus labrax	**SW/BW**	1	1	1	3
Siniperca chuatsi	**FW**	--	1	1	2
Siniperca kneri	**FW**	--	1	1	2
Siniperca scherzeri	**FW**	--	1	1	2
Coreoperca whiteheadi	**FW**	--	1	1	2
Maylandia zebra	**FW/BW**	1	1	--	2
Neolamprologus brichardi	**FW/BW**	1	1	--	2
Pundamilia nyererei	**FW/BW**	1	1	--	2
Haplochromis burtoni	**FW/BW**	1	1	--	2
Oreochromis niloticus	**FW/BW**	1	1	--	2

FW: freshwater, BW: brackish water, SW: sea water.

Supplementary Table 13. Cruel genes and representative neurological pathways in hybrid *S. chuatsi* × *S. scherzeri*

Transcriptome accession no.	Gene symbol	Description	Pathway	log2(SC_W RPKM/SC_X RPKM)	*P*-value	FDR	BMP4-initiated signaling molecules binding sites
**Unigene99889_All**	*adcy3*	Adenylate cyclase 3		1.8454	3.22E-05	9.58E-04	
**Unigene9024_All**	*avt*	Arginine vasotocin/Vasotocin-neurophysin VT 1		-2.331	2.42E-77	1.16E-74	
**Unigene12884_All**	*esr1*	Estrogen receptor alpha	HPG	-1.306	6.58E-12	6.50E-10	
**Unigene50772_All**	*esr1*	Estrogen receptor alpha	HPG	-2.015	3.33E-17	6.83E-15	
**Unigene44248_All**	*fshb*	Follicle-stimulating hormone beta		-1.717	9.05E-43	3.49E-40	
**Unigene88134_All**	*gad*	Glutamate decarboxylase		-12.41	1.34E-06	6.03E-05	
**Unigene25448_All**	*hnmt*	Histamine N-methyltransferase		-2.333	3.07E-13	3.99E-11	Xvent-1
**Unigene95643_All**	*htr1b*	5-hydroxytryptamine (serotonin) receptor 1B	5-HT	1.73	3.16E-05	9.46E-04	
**Unigene5692_All**	*kirrel3*	Kin of IRRE like protein 3		-2.855	9.33E-06	3.34E-04	Xvent-1
**Unigene10121_All**	*npas4*	Neuronal PAS domain-containing protein 4		1.0118	1.06E-08	6.97E-07	
**Unigene51036_All**	*oxt*	Isotocin-neurophysin IT 1		-1.072	4.76E-18	1.02E-15	Xvent-1
**Unigene80547_All**	*rgs6*	Regulator of G-protein signaling 6		-4.402	7.32E-06	2.69E-04	
**Unigene64851_All**	*rgs6*	Regulator of G-protein signaling 6		-2.798	1.73E-05	5.69E-04	
**Unigene27397_All**	*th*	Tyrosine hydroxylase/Tyrosine 3-monooxygenase	Dopamine	1.9508	2.92E-13	3.82E-11	Xvent-1
**Unigene50578_All**	*th*	Tyrosine hydroxylase/Tyrosine 4-monooxygenase	Dopamine	3.265	9.31E-07	4.36E-05	Xvent-1
**Transcriptome accession no.**	**Gene symbol**	**Description**	**Pathway**	**log_2Fold Change (SC_AD/SC_W)**	**P value**	**FDR**	**BMP4-initiated signaling molecules binding sites**
**Unigene1344_All**	*mao*	Amine oxidase [flavin-containing]		1.9346	0.037148		

Supplementary Table 14. Primers used for RT-QPCR of cruel genes

Primer	Sequence (5′-3′)	Product size (bp)	Annealing temperature (°C)	Amplification efficiency (%)
sc-*rpl13a*-F	TATCCCCCCACCCTATGACA	100	60	100.57
sc-*rpl13a*-R	ACGCCCAAGGAGAGCGAACT			
sc-*hnmt*-F	CTACCATCAGCTTCTTCCAGAG	146	57	100.5
sc-*hnmt*-R	AGTGGTCACACACTGACTTATT			
sc-*avt*-F	TCAGAGCAGTAGGGTTAAGAGA	176	55	96
sc-*avt*-R	CCACCAGAGGACAGACTTAGTA			
sc-*maob*-F	GTTCTGAGTTGGTCCGATGTAA	152	57	100.2
sc-*maob*-R	GTTTGAGTGCAGCGAAGTTG			
sc-*rgs6*-F	ACAGTCTCAGAGTCCCATACA	160	55	94.8
sc-*rgs6*-R	GAACTGCTCAGTGTAGCTTATCA			

Supplementary Table 15. Numbers of gill rakers in selected fish species

Species	Numbers of gill rakers
Clupea harengus	56-73
Danio rerio	13-15
Cyprinus carpio	20-25
Ctenopharyngodon idellus	18
Sinocyclocheilus anshuiensis	14
Dicentrarchus labrax	18-29
Larimichthys crocea	8-19
Oreochromis niloticus	30-36
Siniperca chuatsi	6-7
Siniperca kneri	4-7
Siniperca scherzeri	4-6
Coreoperca whiteheadi	7-9
Psammoperca waigiensis	2-7

Supplementary Table 16. Genomic fragments in reporter constructs

Construct name	Sequence (5′- 3′)	Size (bp)
**pGL6-1**	caccttatgcagagtcagttgcatgcgtttttgtgtcacaacatccatcatagtaaatacaggtttcgcttaatatgtgtttttgtccgttctgacagagcagccatcattctctcagcagaact<br>catcactcttcttgttatcctctcctcatcctctctttatcctcttgtcatctaatctcttctcctcacatcctccttttcagtcaggaagcccctggatcgacatcaatgagcctctacaagaagg<br>acggagacagagagaagaaagaaaagagagaggagaatggagaaagagagaaggaaacagactgagagagggacagagaaagagaaagaataaagacagagtgaccaag<br>aaagagaaagaatatggggataaagagaaatgcaaaaatggggacagagggaaagacagagacagagagggagagtaagtaattacaggactcccagtgttttcaaagcccctc<br>ttttatctcacccttcctgtggctgagaaaaggagaagcagcggaaaaggggaggaggaggagaggaagactaaagtccaagctttagtccaaatcagacaacaggcaccagtcat<br>ccacatcacacacacacaaacacagacacactcaaactaatttatagcttctgtttcaaagacatttttatcatctgaaacaaggtgtttgtgtgtgtggggtgggcttcatgtaaagtacttt<br>aatggtgatagttgaagtattgtcagtaatagtaatatgcaacttttctctgtcctttgaatattgtggttattatttatatatgttacatgtctgcctatacgttgtgctgctgacac	834
**pGL6-2**	aatcagacaacaggcaccagtcatccacatcacacacacacaaacacagacacactcaaactaatttatagcttctgtttcaaagacatttttatcatctgaaacaaggtgtttgtgtgtgt<br>ggggtgggcttcatgtaaagtactttaatggtgatagttgaagtattgtcagtaatagtaatatgcaacttttctctgtcctttgaatattgtggttattatttatatatgttacatgtctgcctatac<br>gttgtgctgctgacac	267

Supplementary Table 17. Primers used for knockout and whole mount *in situ* hybridization of zebrafish *edar*

Purpose	Primer	Sequence (5’-3’)
gRNA synthesis	zf-KO-*edar*-F1	TAATACGACTCACTATAGGTGCCGGGCGTCAGGACAGgttttagagctagaaatagc
	zf-KO-*edar*-F2	TAATACGACTCACTATAGGAGAATTTACCAGCCGGACgttttagagctagaaatagc
	zf-KO-universal-R	GGCTGGAGGAGTACTTGATCTC
Mutation identification (nested PCR)	zf-*edar*-outside-F	TGTGTGTGTGTGTGTGTGT
	zf-*edar*-outside-R	CTTGCCATTCAGTTTCCTGTTG
	zf-*edar*-inside-F	CTCGTCTCAGTGAATGTGAGTT
	zf-*edar*-inside-R	GGCATCAATCTGCTCCTCTT
RNA probe synthesis	zf-*edar*-probe-F	TTGAATTCGGCCATAAGAAAGATG
	zf-*edar*-probe-R	TCGGATCCTCTGGCTCACTC
	Anti-sense RNA probe	GCGUCCGUAUCUCAAGGAAGUACGAGAUCUGCCGUCGACACAAAGACUGUGACGC UCUUUACCGCGCCACUGUCCUGACGCCCGGCACCUCUGACAGCGACGCCGAGUGC GGACACUGUUUACCAGGGUACUACAUCCAGGAGAACCGGCCGCAGAACAUCUACG GUAUGGUGUGUCAUUCAUGCCAAAACGCUCCUCGAAACAUCAAAGAGUGCAUGA GGUCCACACCGCCAGCCUCAGGUCGAGCUCCCAGUGUUUCUUCCAGCAGCACCAC UAUAUUCCCUCAGCCAGAGAAAGACCCAACAGGACAGGGUCACCUAGCAACGGCC CUCAUCAUCGCCAUGUCAACCAUCUUCAUCAUGGCCAUCGCUAUAGUGAUGAUCA UCAUGUUCUACAUCCUGAAGAGCAAACCGAGCGGACCAGUCUGCUGUUCUGGUCA GCUCAUCAAAGCUGUAGAAGCUCAGACAAACAUGCAGGAGGAGAAGAAGGAGGC UCAGGAGAACGUGGUGAUCUUUCAGGAGAAGGACGAAUUUGACAAACUCAAGCU UCCAUUCUCCAA

Two gRNA targets located in the fourth coding exon were used in the present study. Targeting sites were indicated with underline.

Supplementary Table 18. Primers used for absolute mRNA expression

Primer	Sequence (5'-3')	Product size (bp)	Annealing temperature (°C)	Amplification efficiency (%)
zf-RT-*eda*-F	GGTCCTACTTGACGGAACATA	101	55	102.2
zf-RT-*eda*-R	GAGTTTTGTCCACCATCACC			
zf-RT-*edar*-F	GCACCACCAACACCATCA	124	55	104.8
zf-RT-*edar*-R	CTCAGACCTTCCGCAACA			
zf-RT-*bmp4*-F	CGAGCCAACACCGTGAG	111	55	101
zf-RT- *bmp4*-R	TGGGATGCTGCTGAGATT			
sc-RT-*eda*-F	TGGGATTATTCCTGCTATCGC	286	58	99.5
sc-RT-*eda*-R	CTTTTCGCTCGCTGAGTTATG			
sc-RT-*edar*-F	GCGTCTGAGAAGCAAGGATT	214	58	99.8
sc-RT-*edar*-R	TGTGATTGGTGCTGGTGATG			
sc-RT-*bmp4*-F	CTGAGGAGAACGGAGCACAT	180	58	98.8
sc-RT-*bmp4*-R	GCTCGTCCTCTGGAATGCTTGT			

References

1. Leggett, R. M., Clavijo, B. J., Clissold, L., Clark, M. D. & Caccamo, M. NextClip: an analysis and read preparation tool for Nextera Long Mate Pair libraries. *Bioinformatics* **30**, 566-568, doi:10.1093/bioinformatics/btt702 (2014).
2. Peng, Y., Leung, H. C., Yiu, S. M. & Chin, F. Y. IDBA-UD: a de novo assembler for single-cell and metagenomic sequencing data with highly uneven depth. *Bioinformatics* **28**, 1420-1428, doi:10.1093/bioinformatics/bts174 (2012).
3. Koren, S. *et al.* Canu: scalable and accurate long-read assembly via adaptive k-mer weighting and repeat separation. *Genome Res.* **27**, 722-736, doi:10.1101/gr.215087.116 (2017).
4. Kajitani, R. *et al.* Efficient de novo assembly of highly heterozygous genomes from whole-genome shotgun short reads. *Genome Res.* **24**, 1384-1395, doi:10.1101/gr.170720.113 (2014).
5. Smit & Hubley. RepeatModeler Open-1.0. <http://www.repeatmasker.org>. (2008-2015).
6. Smit, Hubley & Green. RepeatMasker Open-4.0. <http://www.repeatmasker.org>. (2013-2015).
7. Gotoh, O. A space-efficient and accurate method for mapping and aligning cDNA sequences onto genomic sequence. *Nucleic Acids Res.* **36**, 2630-2638, doi:10.1093/nar/gkn105 (2008).
8. Iwata, H. & Gotoh, O. Benchmarking spliced alignment programs including Spaln2, an extended version of Spaln that incorporates additional species-specific features. *Nucleic Acids Res.* **40**, e161, doi:10.1093/nar/gks708 (2012).
9. Niknafs, Y. S., Pandian, B., Iyer, H. K., Chinnaiyan, A. M. & Iyer, M. K. TACO produces robust multisample transcriptome assemblies from RNA-seq. *Nat. Methods* **14**, 68-70, doi:10.1038/nmeth.4078 (2017).
10. Kim, D., Langmead, B. & Salzberg, S. L. HISAT: a fast spliced aligner with low memory requirements. *Nat. Methods* **12**, 357-360, doi:10.1038/nmeth.3317 (2015).
11. Pertea, M. *et al.* StringTie enables improved reconstruction of a transcriptome from RNA-seq reads. *Nat. Biotechnol.* **33**, 290-295, doi:10.1038/nbt.3122 (2015).
12. Katoh, K. & Standley, D. M. MAFFT multiple sequence alignment software version 7: improvements in performance and usability. *Mol. Biol. Evol.* **30**, 772-780, doi:10.1093/molbev/mst010 (2013).
13. Price, M. N., Dehal, P. S. & Arkin, A. P. FastTree 2--approximately maximum-likelihood trees for large alignments. *PLoS One* **5**, e9490, doi:10.1371/journal.pone.0009490 (2010).
14. Frith, M. C. & Kawaguchi, R. Split-alignment of genomes finds orthologies more accurately. *Genome Biol.* **16**, 106, doi:10.1186/s13059-015-0670-9 (2015).
15. Krzywinski, M. *et al.* Circos: an information aesthetic for comparative genomics. *Genome Res.* **19**, 1639-1645, doi:10.1101/gr.092759.109 (2009).
16. Yang, Z. PAML 4: phylogenetic analysis by maximum likelihood. *Mol. Biol. Evol.* **24**, 1586-1591, doi:10.1093/molbev/msm088 (2007).
17. He, S. *et al.* Insights into food preference in hybrid F1 of *Siniperca chuatsi* (♀) x *Siniperca scherzeri* (♂) mandarin fish through transcriptome analysis. *BMC Genomics* **14**, 601, doi:10.1186/1471-2164-14-601 (2013).
18. Livak, K. J. & Schmittgen, T. D. Analysis of relative gene expression data using real-time quantitative PCR and the $2^{-\Delta\Delta CT}$ method. *Methods* **25**, 402-408 (2001).
