# Supplementary material for: Mandarin fish (Sinipercidae) genomes provide insights into innate predatory feeding
Source: Commun Biol. 2020 Jul 9;3:361. doi: 10.1038/s42003-020-1094-y (PMC7347838; doi:10.1038/s42003-020-1094-y)
Supplement: Supplementary file 3 — Description of Additional Supplementary Files [file 42003_2020_1094_MOESM3_ESM.pdf]

Description of Additional Supplementary Files

File Name: **Supplementary Data 1**
Description: Source Data
