# Supplementary material for: Mandarin fish (Sinipercidae) genomes provide insights into innate predatory feeding
Source: Commun Biol. 2020 Jul 9;3:361. doi: 10.1038/s42003-020-1094-y (PMC7347838; doi:10.1038/s42003-020-1094-y)
Supplement: Supplementary file 4 — Reporting Summary [file 42003_2020_1094_MOESM4_ESM.pdf]

nature research

Corresponding author(s): Xu-Fang Liang & Heiner Kuhl

Last updated by author(s): Apr 20, 2020

Reporting Summary

Nature Research wishes to improve the reproducibility of the work that we publish. This form provides structure for consistency and transparency in reporting. For further information on Nature Research policies, see Authors & Referees and the Editorial Policy Checklist .

Statistics

For all statistical analyses, confirm that the following items are present in the figure legend, table legend, main text, or Methods section.

n/a	Confirmed	
☐	☒	The exact sample size (n) for each experimental group/condition, given as a discrete number and unit of measurement
☐	☒	A statement on whether measurements were taken from distinct samples or whether the same sample was measured repeatedly
☐	☒	The statistical test(s) used AND whether they are one- or two-sided<br>*Only common tests should be described solely by name; describe more complex techniques in the Methods section.*
☒	☐	A description of all covariates tested
☒	☐	A description of any assumptions or corrections, such as tests of normality and adjustment for multiple comparisons
☐	☒	A full description of the statistical parameters including central tendency (e.g. means) or other basic estimates (e.g. regression coefficient) AND variation (e.g. standard deviation) or associated estimates of uncertainty (e.g. confidence intervals)
☒	☐	For null hypothesis testing, the test statistic (e.g. F, t, r) with confidence intervals, effect sizes, degrees of freedom and P value noted<br>*Give P values as exact values whenever suitable.*
☒	☐	For Bayesian analysis, information on the choice of priors and Markov chain Monte Carlo settings
☒	☐	For hierarchical and complex designs, identification of the appropriate level for tests and full reporting of outcomes
☒	☐	Estimates of effect sizes (e.g. Cohen's d, Pearson's r), indicating how they were calculated

Our web collection on statistics for biologists contains articles on many of the points above.

Software and code

Policy information about availability of computer code

Data collection	No software was used.
Data analysis	CANU v1.0, Falcon, LAST, RAGOUT, Quiver, BWA, SSPACE, PBJelly, BLASTn, RAxML, MACSE, TranslatorX, HyPhy, NextClip v1.3.2, IDBA-UD, NEWBLER v3.0, RepeatModeler v1.0.8, RepeatMasker v4.0.6, SPALN v2.06f, TRANSDECODER, TACO, HISAT2, STRINGTIE v1.2.3, MAFFT, FASTTREE2, CS Illustrator 6.0, SPSS 19.0.

For manuscripts utilizing custom algorithms or software that are central to the research but not yet described in published literature, software must be made available to editors/reviewers. We strongly encourage code deposition in a community repository (e.g. GitHub). See the Nature Research guidelines for submitting code & software for further information.

Data

Policy information about availability of data

All manuscripts must include a data availability statement. This statement should provide the following information, where applicable:

- Accession codes, unique identifiers, or web links for publicly available datasets
- A list of figures that have associated raw data
- A description of any restrictions on data availability

The genome assemblies of Siniperca chuatsi, Siniperca scherzeri, Coreoperca whiteheadi and Siniperca kneri have been deposited at http://genomes.igb-berlin.de. The raw sequencing data and genome assemblies have been submitted to NCBI under the Accession: PRJNA513951.

Field-specific reporting

Please select the one below that is the best fit for your research. If you are not sure, read the appropriate sections before making your selection.

Life sciences study design

All studies must disclose on these points even when the disclosure is negative.

Sample size	Sample sizes were listed in Replication Section (below).
Data exclusions	n/a
Replication	1. Section "Gill rakers and food intake of edar-/- zebrafish": 6 biological replicates for measurement of food intake of brine shrimp or dead zebrafish.<br>2. Section "Absolute mRNA expression of eda and edar": Absolute copies of eda and edar in adult S. chuatsi and D. rerio (n = 3 for eda, n = 6 for edar).<br>3. Section "Treatment of activator and inhibitor of edar in zebrafish": In each group (contronl, activin A treatment, BMP4 treatment), larvae were randomly divided into 3 tanks (n = 20).<br>4. Section "DHS mapping": Luciferase activity of pGL6-1 (n = 8); Luciferase assay for pGL6-2 (n = 12); Absolute copies of bmp4 in adult S. chuatsi (n =5), D. rerio (n = 4) and O. niloticus (n = 5).
Randomization	Section "Gill rakers and food intake of zebrafish with activin A and BMP4 treatment": Zebrafish larvae were randomly distributed into three groups: treated with edar activator activin A (R&D Systems, Minneapolis, MN, USA) at a final concentration of 1 ng/ml; treated with edar inhibitor BMP4 (R&D Systems) at 50 ng/ml; control (without treatment).
Blinding	n/a

Reporting for specific materials, systems and methods

We require information from authors about some types of materials, experimental systems and methods used in many studies. Here, indicate whether each material, system or method listed is relevant to your study. If you are not sure if a list item applies to your research, read the appropriate section before selecting a response.

Materials & experimental systems

n/a	Involved in the study	
☒	☐	Antibodies
☐	☒	Eukaryotic cell lines
☒	☐	Palaeontology
☐	☒	Animals and other organisms
☒	☐	Human research participants
☒	☐	Clinical data

Methods

n/a	Involved in the study	
☒	☐	ChIP-seq
☒	☐	Flow cytometry
☒	☐	MRI-based neuroimaging

Eukaryotic cell lines

Policy information about cell lines

Cell line source(s)	Human embryonic kidney 293T (HEK293T) cell line was obtained from China Center for Type Culture Collection (CCTCC, Wuhan, Hubei, China).
Authentication	HEK293T cell line was not authenticated.
Mycoplasma contamination	HEK293T cell line was not tested for mycoplasma.
Commonly misidentified lines (See ICLAC register)	No commonly misidentified cell lines were used.

Animals and other organisms

Policy information about studies involving animals; ARRIVE guidelines recommended for reporting animal research

Laboratory animals	Zebrafish embryos (AB strains) were obtained from China Zebrafish Resource Center (CZRC, Wuhan, Hubei, China).
Wild animals	n/a
Field-collected samples	Mandarin fish S. chuatsi, S. kneri, S. scherzeri and C. whiteheadi inbred lines were obtained from Chinese Perch Research Centre of Huazhong Agricultural University (Wuhan, Hubei, China).
Ethics oversight	The study was approved by the Institutional Animal Care and Use Ethics Committee of Huazhong Agricultural University (Wuhan, Hubei, China). The protocol was approved by the committee on the ethics of animal experiments of the university. All efforts

were made to minimize suffering in animals.

Note that full information on the approval of the study protocol must also be provided in the manuscript.
